# Nuclear Factor-Kappa B and Alzheimer Disease, Unifying Genetic and Environmental Risk Factors from Cell to Humans

**DOI:** 10.3389/fimmu.2017.01805

**Published:** 2017-12-11

**Authors:** Simon Vann Jones, Ilias Kounatidis

**Affiliations:** ^1^Department of Psychiatry, Warneford Hospital, University of Oxford, Oxford, United Kingdom; ^2^Laboratory of Cell Biology, Development and Genetics, Department of Biochemistry, University of Oxford, Oxford, United Kingdom

**Keywords:** nuclear factor-kappa B, Alzheimer, dementia, cell lines, invertebrate models, rodents, humans

## Abstract

Alzheimer’s disease (AD) is the most common form of dementia, an eversible, progressive disease that causes problems with memory, thinking, language, planning, and behavior. There are a number of risk factors associated with developing AD but the exact cause remains unknown. The predominant theory is that excessive build-up of amyloid protein leads to cell death, brain atrophy, and cognitive and functional decline. However, the amyloid hypothesis has not led to a single successful treatment. The recent failure of Solanezumab, a monoclonal antibody to amyloid, in a large phase III trial was emblematic of the repeated failure of anti-amyloid therapeutics. New disease targets are urgently needed. The innate immune system is increasingly being implicated in the pathology of number of chronic diseases. This focused review will summarize the role of transcription factor nuclear factor-kappa B (NF-κB), a key regulator of innate immunity, in the major genetic and environmental risk factors in cellular, invertebrate and vertebrate models of AD. The paper will also explore the relationship between NF-κB and emerging environmental risk factors in an attempt to assess the potential for this transcription factor to be targeted for disease prevention.

## Introduction

The global burden of dementia is devastating, with an estimated 35 million people affected and the annual cost estimated to exceed $1 trillion by 2018 (1, 2). Despite greater knowledge of the pathogenic sequelae of disease, repeated failure in drug trials has led to a switch in emphasis from disease treatment to disease prevention (3–5). Alzheimer’s disease (AD) is the most common dementia subtype yet no single theory has been able to account for the multiple risk factors leading to the pathological and clinical features (3). The deposition of excess extracellular beta amyloid (Aβ) protein and subsequent taupathy has long been felt to be a cause of the disease overshadowing alternative hypotheses including microglial dysfunction, vascular disease, mitochondrial insufficiency, and metabolic disease.

However, the recent failure of another promising anti-amyloid treatment in large phase III trials has dealt a significant blow to the credibility of the amyloid hypothesis (6). The pathognomonic role of Aβ is now also being questioned with the discovery that Aβ acts as an antimicrobial peptide (AMP) in cell lines, nematode, and rodent models. Aβ production following exposure to neurotoxic fungi and bacteria provided significant neuroprotection (7). Amyloid over-production may therefore be a downstream product of immune dysregulation rather than a disease process in itself. In support of this, several genes are involved in innate immunity are associated with an increase in AD (8) (see Table 1). More work is clearly needed to explore the interaction between amyloid and the innate immune system.

**Table 1:** Emerging genetic risk factors for Alzheimer’s disease (AD) and their associated with nuclear factor-kappa B (NF-κB) and amyloid (8).

Gene implicated in late onset AD	Function	Increased risk of AD	Interaction with NF-κB	Interaction with amyloid
TREM2	Immunity	Reduced expression increases risk: slight–medium	NF-κB suppresses hippocampal TREM2 expression (9)	TREM2 required for microglial amyloid clearance (9)
CD33	Immunity	Mild	CD33 activates NF-κB in myeloid cells	CD33 inhibits microglial Aβ uptake and clearance (10)
CR1	Immunity	Mild–medium	Microglial CR1 activation associated with increase in NF-κB (11)	Uncertain (11)
INPP5D	Immunity	Mild	Negative regulator of NF-κB expression (12)	Uncertain

Aging is the most significant risk factor for developing AD and recent findings have shown tissue specific brain inflammation, mediated by NF-κB is associated with aging (12–15). Hypothalamic NF-κB levels are negligible in young mice and *Drosophila*. Significant activation begins in middle age. The resulting downstream increase in AMPs leads to increased local microglial activity, subsequent decline in gonadotrophins and aging (12, 14). Interestingly, hypothalamic inflammation is relatively higher when compared to neurons and glial cells in other vulnerable brain regions such as the hippocampus (12). In support of this hypothalamic-specific regulation of aging, drugs known to extend lifespan in mice reduce hypothalamic inflammation but have little anti-inflammatory effect on hippocampal neurons (14).

**Figure 1:**
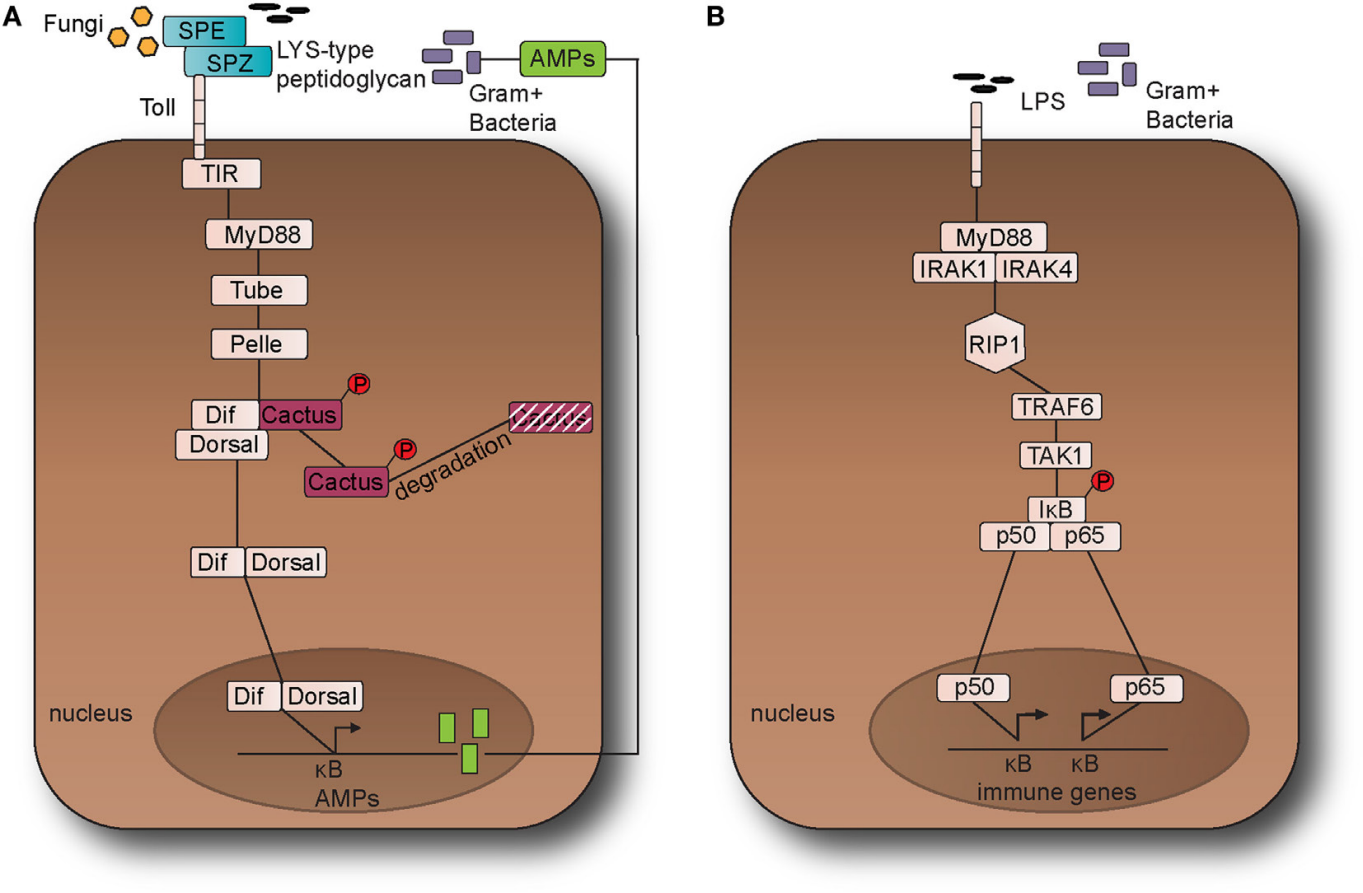
The Toll pathway in fruit fly and the Toll-like receptor (TLR) 4 pathway in the mouse. **(A)** The Toll pathway in *Drosophila melanogaster* detects Gram-positive bacteria and fungi is activated through an endogenous ligand, namely Nerve Growth Factor-related cytokine Spaetzle (SPZ) which is processed by Spaetzle-processing enzyme (SPE). Toll receptor activation results in the recruitment of the adaptor proteins namely myeloid differentiation primary response 88 (dMyD88), Tube, and Pelle, which promotes signaling to Cactus and its ankyrin-repeat domains. Cactus is bound to the nuclear factor-kappa B (NF-κB) transcription factors dorsal-related immunity factor (DIF) and Dorsal and following activation of the pathway, it is phosphorylated and degraded. The above signaling events result in the nuclear translocation of DIF or Dorsal that stimulate the transcriptional upregulation of antimicrobial peptide (AMP) genes, such as Drosomycin. **(B)**. TLR4 receptor in *M. musculus* detects lipopolysaccharides (LPS) from Gram-negative bacteria. Myeloid differentiation primary response 88 (MyD88) is recruited with Interleukin-1 receptor-associated kinases 1 and 4 (IRAK1, IRAK4), receptor-interacting protein 1 (RIP1), and tumor necrosis factor (TNF) receptor-associated factor 6 (TRAF6). TRAF6 is self-ubiquitinated in order to recruit transforming growth factor beta (TGF-β) activated kinase 1 (TAK1) and TAK1-associated binding proteins 1 and 2 (TAB1 and TAB2). The latter leads to the activation of the IκB kinase (IKK) complex that in turns phosphorylates the inhibitor of NF-κB (IκB). This leads to the release of NF-κB that translocates to the nucleus and initiates the transcriptional induction of inflammatory and immune response related genes. This leads to the translocation of the NF-κB transcription factors p50 and p65 to the nucleus, which in turns initiates the transcriptional induction of inflammatory and immune response related genes.

Known inducers of NF-κB activity are highly variable and include reactive oxygen species (ROS), interleukin 1-beta (IL-1β), tumor necrosis factor alpha (TNF-α), bacterial lipopolysaccharides (LPS), isoproterenol, and ionizing radiation (16, 17). In addition to stimuli that activate NF-κB in other tissues, NF-κB in the nervous system can be activated by growth factors and synaptic transmission such as glutamate (18). These activators of NF-κB in the nervous system all converge upon the inhibitor of kinase kinase (IKK) complex (Figure 1).

**Figure 2:**
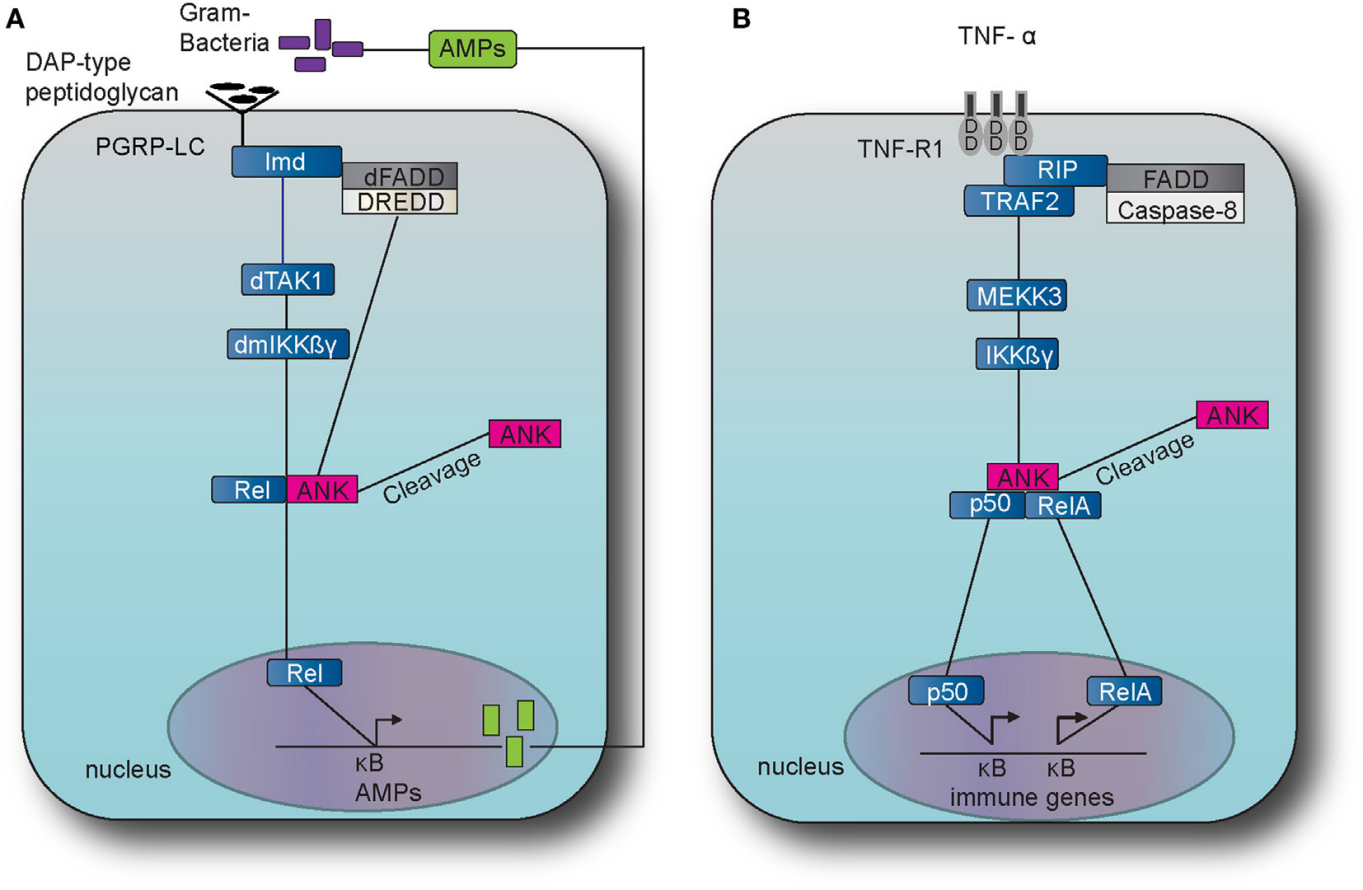
The immune deficiency (IMD) pathway in fruit fly and the TNF pathway in the mouse. **(A)** The IMD pathway in *Drosophila melanogaster* is activated by Gram-negative bacteria and certain Gram-positive bacilli. The intracellular adaptor protein immune deficiency (Imd) interacts with the *Drosophila* Fas-associated death domain (dFADD) and the death-related ced-3/Nedd2-like caspase (DREDD) that cleaves Imd, which is then activated by K63 ubiquitination. This leads to the activation of the transforming growth factor beta activated kinase 1 (TAK1) that in turn activates the *D. melanogaster* inhibitor of kinase kinase ß and γ complex (dmIKKßγ). Activation results in the translocation of the nuclear factor-kappa B (NF-κB) transcription factor Relish (Rel) Dorsal to the nucleus which induces the transcription of antimicrobial peptide (AMP) genes, such as Diptericin. **(B)** The tumor necrosis factor (TNF) pathway in *M. musculus* is activated by TNF alpha (TNF-α) which binds and activates the transmembrane receptors R1 (TNFR1) and recruits the receptor-interacting protein (RIP) and TNF receptor-associated factor 2 (TRAF2). TRAF2 employs mitogen-activated protein kinase kinase kinase 3 (MEKK3) which in turn activates the inhibitor of kinase kinase ß and γ complex (IKKßγ), which results to the translocation of NF-κB transcription factors p50 and Rel A. The latter translocation induces expression of several genes that are involved to immunity and inflammation.

Nuclear factor-kappa B transcription factors include a collection of proteins with functions conserved from the fruit fly *Drosophila melanogaster* to rodents and to humans (Figures 1 and 2). They are present in all human and most animal cells and regulate the expression of more than 400 genes, including a multitude of inflammatory mediators associated with a variety of chronic inflammatory diseases including cancer, diabetes, and AD (19, 20). Lately, Rel/NF-κB homologs have also been found to occur even in organisms as simple as *Cnidarians* (e.g., sea anemones and corals), Porifera (sponges), and the single-celled eukaryote *Capsaspora owczarzaki*, but are notably absent in yeast and the nematode *Caenorhabditis elegans* (21). Activated NF-κB regulates the expression of specific genes, including isoforms of SET, directly implicated in the pathogenesis of AD (22). Conversely, expression of the mammalian family of Sirtuin deacetylases, known to attenuate the effects of aging, down regulates NF-κB (23, 24).

Studies of aging populations have enabled the identification of a number of genetic and environmental risk factors that appear to influence susceptibility to developing AD (25, 26). This paper will review the interaction between these risk factors and NF-κB, first looking at the major known genetic risk factors in cell, invertebrate, and vertebrate models. It will then focus on the major known environmental risk factors in these models before reviewing emerging environmental risk factors. Finally, the paper will review protective mechanisms across various experimental models and whether their association with NF-κB.

The repeated failure of disease modifying trials in AD demands that new treatment targets are urgently identified. The recent findings that implicate the innate immune system in AD provides an opportunity to review the evidence for NF-κB as a key immune system regulator in the prodromal stage in the hope of identifying a target for treatment and prevention.

## Genetics

### Neuronal Cell Lines/Human Autopsy Studies

Overexpression of the amyloid precursor protein (APP) gene is associated with familial aggregation of late onset AD and dramatically increases susceptibility to early AD in Down’s syndrome. APP is cleaved by the beta-secreatase BACE1 into amyloid monomers that form oligomers that eventually become plaques in the brain and vasculature. Both BACE1 and NF-κB are increased in the brains of AD patients, with NF-κB directly upregulating BACE1 and the APP gene (27, 28). Medications such as minocycline, that inhibit NF-κB but not BACE1 or APP, reverse this process (28, 29).

The e4 variant of the APOE gene, which codes for a cholesterol transporting protein, is the largest, single gene risk factor for AD (30). In APOE e4-positive Schwann cell lines, when compared to APOE e3-expressing cells, excess production of IL6, IL10, and nitrous oxide results from a failure to inhibit NF-κB (31). These findings are replicated in neural cells and fibroblasts from AD patients where APOE e4 acts as a transcription factor responsible for regulating NF-κB expression (32). Curiously, cells from the somatosensory cortex of AD patients, an area of the brain that is resistant to disease, display upregulation of NF-κB (33). However, this may reflect the earliest inflammatory hallmark of disease as previous autopsy studies have shown increased NF-κB activation in evolving Aβ deposits with a reduction in areas surrounding more mature plaques (19, 33).

### Invertebrate Models

A genetic screen for dominant suppressors and enhancers in a *Drosophila* model of Aβ-driven neurodegeneration revealed that Toll gene (receptor of Drosophila toll pathway) and key downstream components (*dif, pelle, cactus*), play a central role in mediating the neuropathological activities (34). Conversely, genetic overexpression results in accelerated deterioration of the phenotype suggesting that NF-κB significantly enhances the pathological potential of Aβ (35).

Genetic suppression of the immune deficiency (IMD) NF-κB pathway in glial cells in a *Drosophila* model of early onset neurodegeneration dramatically rescues brain pathology, reduced activity, and short lifespan (13). Genetic overexpression of NF-κB pathways at neuronal or glial tissue level leads to phenotypes resembling AD models with locomotor disability, accelerated neurodegeneration, and premature mortality (13).

### Vertebrate Models

Age is the biggest risk factor for dementia and systemic inflammation increases as animal’s age, a process known as inflammaging (36). Microglia priming in mice induces a highly conserved transcriptional signature with aging characterized by NF-κB expression and neuronal death (37). In rats, NF-κB expression increases in normal aging leading to production of neurodegenerative pro-inflammatory enzymes COX-2 and iNOS (38). These changes are reversed by suppression of brain NF-κB activation using the anti-inflammatory *Lactobacillus pentosus* var. plantarum C29, restoring brain-derived neurotrophic factor (BDNF) levels and memory (39). In mice, the observed positive correlation between NF-κB activity and neuronal apoptosis suggests a role of NF-κB in hippocampal neuroapoptosis (40). Supporting this, NF-κB induces pro-apoptotic increases in TNF and iNOS in the hippocampus of rats exposed to neurotoxin (41).

Inactivating specific Sirtuin anti-aging genes in mice results in chronic NF-κB overexpression leading to accelerated aging and dramatically reduced lifespan (23). In Sirtuin replete models, overexpression *via* biofeedback dysregulation results in premature aging through chronic production of excessive ROS, leading to telomere dysfunction, cellular senescence, and premature death (42). Age-related NF-κB activation feeds into a positive feedback loop in microglial cells causing perpetual inflammation and multiple brain responses including epigenetic suppression of GnRH genes in the hypothalamus (14). Microglial-derived NF-κB-TNF-α axis plays a key role in homeostatic synaptic scaling, a form of synaptic plasticity. However, overexpression results in disrupted neuronal networks and behavior mimicking obsessive–compulsive disorder (OCD) (43, 44). Suppressing this pathway mediates some of the OCD-like behavioral problems in mouse models of frontotemporal dementia (44). Specifically, under-expression of NF-κB in the mouse brain results in delayed onset of age-related pathology across all organ systems *via* preservation of the hypothalamic–pituitary–adrenal axis and GnRH levels (14).

## Environment

### Neuronal Cell Lines/Human Autopsy Studies

Type 2 diabetes mellitus (T2DM), a metabolic condition characterized by a decrease in sensitivity to endogenous insulin, is the best established environmental risk factor for the development of AD, increasing relative risk by 50% (45). Diabetes induces Aβ pathology *via* NF-κB upregulation and independent overexpression of BACE1 (46, 47). Inflammatory mediators are known to contribute to insulin resistance creating a pro-inflammatory feedback loop in diabetes (48). Administering advanced glycation end products that mimic diabetic driven pathology results in elevated BACE1 and consequent NF-κB overexpression in both rat brains *in vivo* and neuroblastoma cells lines (49). NF-κB suppression using Adiponectin rescues Aβ pathology in human T2DM neuroblastoma cells (50). Similarly, leukotriene D4, an inflammatory signaling molecule elevated in metabolic disorders, induces Aβ synthesis in primary neurons at 24 h with increases in NF-κB seen after just 1 h (51, 52). Treatment of the culture with NF-κB inhibitor pyrrolidine dithiocarbamate (PDTC) inhibited Aβ generation with down regulation of Aβ generating beta- and gamma-secretase activity suggesting NF-κB regulates Aβ synthesis in metabolic disease (52).

In human neuroblastoma cells, the metabolic enzyme protein arginine methyltransferase 5 (PRMT5) regulates cellular metabolism, protecting the cell in times of stress. Aβ downregulates this process leading to NF-κB overexpression, metabolic dysfunction, and premature cell death (53). Inhibiting NF-κB reduces apoptosis and Aβ deposits even in a metabolically dysfunctional organism or human cell lines suggesting a gatekeeper role for NF-κB in maintaining metabolic homeostasis and subsequent neuroprotection independent of PRMT5 activity (53).

### Invertebrate Models

In *Drosophila* models of chronic diseases including AD, the innate immune system has been identified as a key mediator of neurodegeneration (13, 34, 54, 55). NF-κB overexpression in the fat body cells, analogous to the metabolic syndrome in humans, results in more severe neurodegeneration (56).

Supporting the role of NF-κB in connecting whole-body metabolism with brain health, NF-κB overexpression in the hypothalamus-like pars intercerebralis neurons in *Drosophila* results in overnutrition, impaired metabolic learning, poor memory consolidation, and metabolic disorder characterized by increased lipid levels and shortened lifespan (57). Conversely, genetic knockdown of NF-κB signaling in glial cells leads to elevated adipokinetic and glucagon-like hormone levels, reduced glucose and lipid levels, and extension of “healthspan” (13).

### Vertebrate Models

In rodent and primate models, diabetes and obesity drive overexpression of NF-κB in the hypothalamus creating a destructive feedback loop where further NF-κB expression promotes hypertension, overnutrition, and decreased insulin sensitivity (58–61). Injecting Aβ into the brains of mice and macaques results in an increase in NF-κB in the cell nuclei of the hypothalamus and subsequent induction of peripheral glucose intolerance (62). In this model, pharmacological inhibition of NF-κB maintained peripheral metabolic homeostasis. Inducing diabetes in rats results in hippocampal NF-κB-dependent neurodegeneration *via* disruption of CREB phosphorylation, reducing levels of protective downstream proteins including BDNF (63).

The tetracycline derivative Minocycline inhibits NF-κB and prevents further Aβ deposition in a mouse model of diabetes-driven AD. BACE1 activity remained elevated demonstrating an NF-κB-dependent protective mechanism (29). Mice fed on high-fat diets demonstrate elevated brain BACE1 expression as do transgenic diabetic mice. Administration of the anti-inflammatory agent all-trans-retinoic acid reduces BACE1 expression in both WT and mutant but this effect is abolished when the NF-κB-binding site at the promoter region of BACE1 is mutated (64).

## Emerging Risk Factors

### Alcohol Intake

In *Drosophila*, alcohol consumption activates Toll-NF-κB signaling increasing ethanol resistance and gene products known to be outputs of innate immune signaling are rapidly induced following ethanol exposure (65). Ethanol treatment of cultured hippocampal rat neurons causes a dose- and time-dependent increase in NF-κB-DNA-binding activity, resulting in significant upregulation of inflammatory markers and increased susceptibility to neurotoxins; reversible by applying NF-κB inhibitors (66, 67).

Opposing, the consumption of moderate amounts of alcohol, particularly red wine, is associated with a reduced risk of AD. Anthocyanin, a polyphenol found in wines, protects rat hippocampal neurons against oxidative stress *via* NF-κB suppression (41, 68).

### Sleep

Sleep quality and well-being are symbiotic and reduced sleep quality is increasingly being associated with increased risk of dementia. Sleep–wake cycle homeostasis is important in the processing and removal of Aβ plaques which, in turn, are known to dysregulate this reparative process (69). Sleep disruption and deprivation are known to cause over expression of the NF-κB pathway in hippocampal cell cultures, fruit flies, rodents, and humans (70–73). Improvement in sleep quality in older adults is correlated with a reduction in circulating NF-κB (74).

### Traumatic Brain Injury (TBI)

Traumatic brain injury activates both microglia and astrocytes and induces self-sustaining inflammatory responses in the brain *via* NF-κB activation (75, 76). In *Drosophila*, flies with TBI exhibited temporary incapacitation, ataxia, activation of the innate immune response, neurodegeneration, and death similar to humans with TBI (77, 78). Rat models have demonstrated the acute onset and prolonged overexpression of NF-κB in brain regions most commonly associated with post-injury atrophy (79, 80). Recent studies have shown NF-κB to be significantly elevated in the ipsi-lateral cortex of both adult and old TBI mice in a time-dependent manner (81). These interactions started immediately post-injury in the old mice compared to the adult mice suggesting an age-related failure of NF-κB suppression.

## Protective Factors

### Exercise

Under expressing NF-κB in mice results in greater endurance, cognitive performance, and resistance to obesity (82). An aerobic exercise protocol in wild-type rats attenuated age-related memory decline and decreased hippocampal NF-κB levels and atrophy (83). In rats fed a pro-inflammatory diet and subjected to either strength training, aerobic exercise or a combination of both, all protocols reduced liver and muscle NF-κB levels to pre-diet levels (84). These findings are supported by studies demonstrating the indirect suppression of NF-κB *via* cytokine IL-10, a potent NF-κB inhibitor, induced by exercise (85).

### Diet

Curcumin, a constituent of turmeric, has gained much attention in recent years for its potential as a neuroprotective compound. In a *Drosophila* model of neurodegeneration, the curcumin analog C150 significantly reduced neuronal cell death, increased healthy lifespan, and reduced DNA mutation in brain tissue by suppressing NF-κB (86).

The neuroprotective effects resulting from the Mediterranean diet or those rich in oily fish may be mediated *via* the anti-inflammatory properties of key nutrients (87). Aβ induced increases in the translocation NF-κB subunits is attenuated in the presence of tyrosol (Tyr) and hydroxytyrosol (OH-Tyr) found abundantly in olive oil (88). Transgenic increases in omega-3 polyunsaturated fatty acid in the brain of mice reduces the inflammatory response to LPS challenge *via* NF-κB pathways (89).

The omega 3 fatty acid eicosapentaenoic acid indirectly downregulates NF-κB expression acting as a ligand at peroxisome proliferator-activated receptor gamma, a regulator of fatty acid storage and glucose metabolism, and reduces symptoms of depression (87, 90). In AD-mouse hippocampal slices, food-derived anti-oxidants provide neuroprotection and reduce Aβ *via* the anti-inflammatory properties of the polyphenolic compounds within them (91). Specifically, the phenolic compound resveratrol reduces Aβ-induced migroglial activity and neuroinflammation *via* NF-κB suppression in murine microglial and macrophage cells (92).

### Anti-inflammatory Drugs

The prolonged use of non-steroidal anti-inflammatory drugs (NSAIDs) is associated with a reduction in the AD risk (93). In primary rat, neurons and human neuronal cell lines NSAIDs strongly inhibit NF-κB-driven expression of BACE1 activity preventing the cleavage of Aβ from APP (28, 94). Long-term administration of potent NSAID indomethacin blocks activation of NF-κB and significantly reduced the amyloid pathology in transgenic AD mice (95).

Aspirin, an NSAID derived from salicylic acid, completely inhibits Aβ activation of the NF-κB pathway, reducing levels of pro-inflammatory cytokines and chemokines, and increasing levels of anti-inflammatory IL-10 in rodent microglia and neurons resulting in recruitment of Aβ phagocytic microglia and improved cognitive and synaptic functioning (28, 96).

## Conclusion

Epidemiological studies are beginning to converge of common risk factors for the development of AD with strong signals also emerging for certain protective factors. The emergence of NF-κB as a regulator of aging and proliferation of studies implicating NF-κB over-activation in a number of neurodegenerative diseases suggests that it may be important in modulating the risk of disease. This review has highlighted the intimate relationship between all known and emerging risk and protective factors for Alzheimer’s and NF-κB activity, implicating over-activation with an increased risk of the disease and suppression being associated with risk reduction. Future work, both in models of disease and trials in man, should focus on therapies that directly target NF-κB overexpression to explore whether early risk identification and targeted anti-inflammatory treatment can significantly increase the time of disease onset and, consequently, reduce the incidence of this devastating disease.

## Author Contributions

SJ and IK wrote the manuscript, and IK designed the figures.

## Conflict of Interest Statement

The authors declare that the research was conducted in the absence of any commercial or financial relationships that could be construed as a potential conflict of interest.

## References

[1] Wimo A Jönsson L Bond J Prince M Winblad B International AD The worldwide economic impact of dementia 2010. Alzheimer Dement (2013) 9(1):1–11. 10.1016/j.jalz.2012.11.006 23305821

[2] Wimo A Guerchet M Ali G-C Wu Y-T Prina AM Winblad B The worldwide costs of dementia 2015 and comparisons with 2010. Alzheimers Dement (2017) 13(1):1–7. 10.1016/j.jalz.2016.07.150 27583652 PMC5232417

[3] Jack CR Knopman DS Jagust WJ Shaw LM Aisen PS Weiner MW Hypothetical model of dynamic biomarkers of the Alzheimer’s pathological cascade. Lancet Neurol (2010) 9(1):119–28. 10.1016/S1474-4422(09)70299-6 20083042 PMC2819840

[4] Imtiaz B Tolppanen AM Kivipelto M Soininen H. Future directions in Alzheimer’s disease from risk factors to prevention. Biochem Pharmacol (2014) 88(4):661–70. 10.1016/j.bcp.2014.01.003 24418410

[5] Gauthier S Albert M Fox N Goedert M Kivipelto M Mestre-Ferrandiz J Why has therapy development for dementia failed in the last two decades? Alzheimer Dement (2016) 12(1):60–4. 10.1016/j.jalz.2015.12.003 26710325

[6] Hawkes N Promise of new Alzheimer’s drug is dashed after lack of evidence. BMJ (2016) 355:i6362 10.1136/bmj.i6362 27884817

[7] Vijaya Kumar DK Choi SH Washicosky KJ Eimer WA Tucker S Ghofrani J Amyloid-β peptide protects against microbial infection in mouse and worm models of Alzheimer’s disease. Sci Transl Med (2016) 8(340):340ra72 10.1126/scitranslmed.aaf1059 PMC5505565 27225182

[8] Robinson M Brenda Y Lee A Francis T Hane J Recent progress in Alzheimer’s disease research, part 2: genetics and epidemiology. Alzheimer Dement (2017) 57(2):317–30. 10.3233/JAD-161149 PMC5366246 28211812

[9] Zhao Y Bhattacharjee S Jones BM Dua P Alexandrov PN Hill JM Regulation of TREM2 expression by an NF-κB-sensitive miRNA-34a. Neuroreport (2013) 24(6):318–23. 10.1097/WNR.0b013e32835fb6b0 23462268 PMC4072209

[10] Griciuc A Serrano-Pozo A Parrado AR Lesinski AN Asselin CN Mullin K Alzheimer’s disease risk gene CD33 inhibits microglial uptake of amyloid beta. Neuron (2013) 78(4):631–43. 10.1016/j.neuron.2013.04.014 23623698 PMC3706457

[11] Zhu XC Yu JT Jiang T Wang P Cao L Tan L CR1 in Alzheimer’s disease. Mol Neurobiol (2015) 51(2):753–65. 10.1007/s12035-014-8723-8 24794147

[12] Li X Long J He T Belshaw R Scott J Integrated genomic approaches identify major pathways and upstream regulators in late onset Alzheimer’s disease. Sci Rep (2015) 5:12393 10.1038/srep12393 26202100 PMC4511863

[13] Kounatidis I Chtarbanova S Cao Y Hayne M Jayanth D Ganetzky B NF-κB immunity in the brain determines fly lifespan in healthy aging and age-related neurodegeneration. Cell Rep (2017) 19(4):836–48. 10.1016/j.celrep.2017.04.007 28445733 PMC5413584

[14] Sadagurski M Cady G Miller RA. Anti-aging drugs reduce hypothalamic inflammation in a sex-specific manner. Aging Cell (2017) 16(4):652–6602017. 10.1111/acel.12590 28544365 PMC5506421

[15] Kaltschmidt B Sparna T Kaltschmidt C Activation of NF-κB by reactive oxygen intermediates in the nervous system. Antioxid Redox Signal (1999) 1(2):129–44. 10.1089/ars.1999.1.2-129 11228742

[16] Kounatidis I Ligoxygakis P. *Drosophila* as a model system to unravel the layers of innate immunity to infection. Open Biol (2012) 2(5):120075. 10.1098/rsob.120075 22724070 PMC3376734

[17] Wang L Kounatidis I Ligoxygakis P. *Drosophila* as a model to study the role of blood cells in inflammation, innate immunity and cancer. Front Cell Infect Microbiol (2014) 3:113. 10.3389/fcimb.2013.00113 24409421 PMC3885817

[18] Meffert MK Chang JM Wiltgen BJ Fanselow MS Baltimore D NF-κB functions in synaptic signalling and behaviour. Nat Neurosci (2003) 6(10):1072–8. 10.1038/nn1110 12947408

[19] Ahn KS Aggarwal BB Transcription factor NF-κB: a sensor for smoke and stress signals. Ann N Y Acad Sci (2005) 1056(1):218–33. 10.1196/annals.1352.026 16387690

[20] Karunaweera N Raju R Gyengesi E Münch G Plant polyphenols as inhibitors of NF-κB induced cytokine production – a potential anti-inflammatory treatment for Alzheimer’s disease? Front Mol Neurosci (2015) 8:24 10.3389/fnmol.2015.00024 26136655 PMC4468843

[21] Gilmore T Rel/NF-κB Transcription Factors. Boston University (2017). Available from: www.nf-kb.org. 2017

[22] Feng Y Li X Zhou W Lou D Huang D Li Y Regulation of set gene expression by nfkb. Mol Neurobiol (2016) 54(6):4477–85. 10.1007/s12035-016-9967-2 27351675

[23] Kawahara TL Michishita E Adler AS Damian M Berber E Lin M SIRT6 links histone H3 lysine 9 deacetylation to NF-κB-dependent gene expression and organismal life span. Cell (2009) 136(1):62–74. 10.1016/j.cell.2008.10.052 19135889 PMC2757125

[24] Natoli G When sirtuins and NF-κB collide. Cell (2009) 136(1):19–21. 10.1016/j.cell.2008.12.034 19135883

[25] Reitz C Brayne C Mayeux R. Epidemiology of Alzheimer disease. Nat Rev Neurol (2011) 7(3):137–52. 10.1038/nrneurol.2011.2 21304480 PMC3339565

[26] Sindi S Mangialasche F Kivipelto M Advances in the prevention of Alzheimer’s disease. F1000Prime Rep (2015) 7:50 10.12703/P7-50 26097723 PMC4447057

[27] Grilli M Ribola M Alberici A Valerio A Memo M Spano P Amyloid precursor protein (APP) gene expression is controlled by a NFkB/Rel related protein. In: Hanin I Yoshida M Fisher A, editors. Alzheimer’s and Parkinson’s Diseases Part of the Advances in Behavioral Biology Book Series ABBI. (Vol. 44). US: Springer (1995). p. 105–10.

[28] Chen CH Zhou W Liu S Deng Y Cai F Tone M Increased NF-κB signalling up-regulates BACE1 expression and its therapeutic potential in Alzheimer’s disease. Int J Neuropsychopharmacol (2012) 15(1):77–90. 10.1017/S1461145711000149 21329555

[29] Cai Z Zhao Y Yao S Zhao B Increases in β-amyloid protein in the hippocampus caused by diabetic metabolic disorder are blocked by minocycline through inhibition of NF-κB pathway activation. Pharmacol Rep (2011) 63(2):381–91. 10.1016/S1734-1140(11)70504-7 21602593

[30] Liu C-C Kanekiyo T Xu H Bu G. Apolipoprotein E and Alzheimer disease: risk, mechanisms, and therapy. Nat Rev Neurol (2013) 9(2):106–18. 10.1038/nrneurol.2012.263 23296339 PMC3726719

[31] Zhang KJ Zhang HL Zhang XM Zheng XY Quezada HC Zhang D Apolipoprotein E isoform-specific effects on cytokine and nitric oxide production from mouse Schwann cells after inflammatory stimulation. Neurosci Lett (2011) 499(3):175–80. 10.1016/j.neulet.2011.05.050 21651961

[32] Theendakara VP Peters-Libeu C Bredesen DE Rao RV Novel transcriptional role of Apo e4. Alzheimers Dement (2016) 12(7):224 10.1016/j.jalz.2016.06.400

[33] Conejero-Goldberg C Hyde TM Chen S Herman MM Kleinman JE Davies P Cortical transcriptional profiles in APOE4 carriers with Alzheimer’s disease: patterns of protection and degeneration. J Alzheimer Dis (2015) 48(4):969–78. 10.3233/JAD-150345 26444771

[34] Tan L Schedl P Song H-J Garza D Konsolaki M The *Toll*→NF-κB signaling pathway mediates the neuropathological effects of the human Alzheimer’s Aβ42 polypeptide in *Drosophila*. PLoS One (2008) 3(12):e3966 10.1371/journal.pone.0003966 19088848 PMC2597734

[35] Sarantseva S Timoshenko S Bolshakova O Karaseva E Rodin D Schwarzman AL Apolipoprotein E-mimetics inhibit neurodegeneration and restore cognitive functions in a transgenic *Drosophila* model of Alzheimer’s disease. PLoS One (2009) 4(12):e8191 10.1371/journal.pone.0008191 19997607 PMC2782140

[36] Franceschi C Capri M Monti D Giunta S Olivieri F Sevini F Inflammaging and anti-inflammaging: a systemic perspective on aging and longevity emerged from studies in humans. Mech Ageing Dev (2007) 128(1):92–105. 10.1016/j.mad.2006.11.016 17116321

[37] Holtman IR Raj DD Miller JA Schaafsma W Yin Z Brouwer N Induction of a common microglia gene expression signature by aging and neurodegenerative conditions: a co-expression meta-analysis. Acta Neuropathol Commun (2015) 3:31. 10.1186/s40478-015-0203-5 26001565 PMC4489356

[38] Kim MK Chung SW Kim DH Kim JM Lee EK Kim JY Modulation of age-related NF-κB activation by dietary zingerone via MAPK pathway. Exp Gerontol (2010) 45(6):419–26. 10.1016/j.exger.2010.03.005 20211236

[39] Jeong JJ Woo JY Kim KA Han MJ Kim DH Lactobacillus pentosus var. plantarum C29 ameliorates age-dependent memory impairment in Fischer 344 rats. Lett Appl Microbiol (2015) 60(4):307–14. 10.1111/lam.12393 25598393

[40] Niu Y-L Zhang W-J Wu P Liu B Sun G-T Yu D-M Expression of the apoptosis-related proteins caspase-3 and NF-κB in the hippocampus of Tg2576 mice. Neurosci Bull (2010) 26(1):37–46. 10.1007/s12264-010-6122-3 20101271 PMC5560375

[41] Rehman SU Shah SA Ali T Chung JI Kim MO. Anthocyanins reversed d-galactose-induced oxidative stress and neuroinflammation mediated cognitive impairment in adult rats. Mol Neurobiol (2017) 54(1):255–71. 10.1007/s12035-015-9604-5 26738855

[42] Jurk D Wilson C Passos JF Oakley F Correia-Melo C Greaves L Chronic inflammation induces telomere dysfunction and accelerates ageing in mice. Nat Commun (2014) 2:4172. 10.1038/ncomms5172 24960204 PMC4090717

[43] Stellwagen D Malenka RC. Synaptic scaling mediated by glial TNF-alpha. Nature (2006) 440(7087):1054–9. 10.1038/nature04671 16547515

[44] Krabbe G Minami SS Etchegaray JI Taneja P Djukic B Davalos D Microglial NFκB-TNFα hyperactivation induces obsessive–compulsive behavior in mouse models of progranulin-deficient frontotemporal dementia. Proc Natl Acad Sci U S A (2017) 114(19):5029–34. 10.1073/pnas.1700477114 28438992 PMC5441749

[45] Li X Song D Leng SX Link between type 2 diabetes and Alzheimer’s disease: from epidemiology to mechanism and treatment. Clin Interv Aging (2015) 10:549 10.2147/CIA.S74042 25792818 PMC4360697

[46] Vassar R Kuhn P-H Haass C Kennedy ME Rajendran L Wong PC Function, therapeutic potential and cell biology of BACE proteases: current status and future prospects. J Neurochem (2014) 130(1):4–28. 10.1111/jnc.12715 24646365 PMC4086641

[47] Jonsson T Atwal JK Steinberg S Snaedal J Jonsson PV Bjornsson S A mutation in APP protects against Alzheimer’s disease and age-related cognitive decline. Nature (2012) 488(7409):96–9. 10.1038/nature11283 22801501

[48] Shoelson SE Lee J Goldfine AB Inflammation and insulin resistance. J Clin Invest (2006) 116(7):1793–801. 10.1172/JCI29069 16823477 PMC1483173

[49] Guglielmotto M Aragno M Tamagno E Vercellinatto I Visentin S Medana C AGEs/RAGE complex upregulates BACE1 via NF-κB pathway activation. Neurobiol Aging (2012) 33(1):.e13–27. 10.1016/j.neurobiolaging.2010.05.026 20638753

[50] Chan K-H Lam KS-L Cheng O-Y Kwan JS-C Ho PW-L Cheng KK-Y Adiponectin is protective against oxidative stress induced cytotoxicity in amyloid-beta neurotoxicity. PLoS One (2012) 7(12):e52354. 10.1371/journal.pone.0052354 23300647 PMC3531475

[51] Long EK Hellberg K Foncea R Hertzel AV Suttles J Bernlohr DA Fatty acids induce leukotriene C_4_ synthesis in macrophages in a fatty acid binding protein-dependent manner. Biochim Biophys Acta (2013) 1831(7):1199–207. 10.1016/j.bbalip.2013.04.004 24046860

[52] Wang XY Tang SS Hu M Long Y Li YQ Liao MX Leukotriene D4 induces amyloid-β generation via CysLT 1 R-mediated NF-κB pathways in primary neurons. Neurochem Int (2013) 62(3):340–7. 10.1016/j.neuint.2013.01.002 23318673

[53] Quan X Yue W Luo Y Cao J Wang H Wang Y The protein arginine methyltransferase PRMT5 regulates Aβ-induced toxicity in human cells and *Caenorhabditis elegans* models of Alzheimer’s disease. J Neurochem (2015) 134(5):969–77. 10.1111/jnc.13191 26086249

[54] Petersen AJ Wassarman DA. *Drosophila* innate immune response pathways moonlight in neurodegeneration. Fly (2012) 6(3):169–72. 10.4161/fly.20999 22864563

[55] Lim Y-M Tsuda L. Ebi, a *Drosophila* homologue of TBL1, regulates the balance between cellular defense responses and neuronal survival. Am J Neurodegener Dis (2016) 5(1):62–8. 27073743 PMC4788732

[56] Lim Y-M Yagi Y Tsuda L. Cellular defense and sensory cell survival require distinct functions of *ebi* in *Drosophila*. PLoS One (2015) 10(11):e0141457. 10.1371/journal.pone.0141457 26524764 PMC4629896

[57] Zhang Y Liu G Yan J Zhang Y Li B Cai D. Metabolic learning and memory formation by the brain influence systemic metabolic homeostasis. Nat Commun (2015) 6:6704. 10.1038/ncomms7704 25848677 PMC4391062

[58] Zhang X Zhang G Zhang H Karin M Bai H Cai D Hypothalamic IKKβ/NF-κB and ER stress link overnutrition to energy imbalance and obesity. Cell (2008) 135(1):61–73. 10.1016/j.cell.2008.07.043 18854155 PMC2586330

[59] Posey KA Clegg DJ Printz RL Byun J Morton GJ Vivekanandan-Giri A Hypothalamic proinflammatory lipid accumulation, inflammation, and insulin resistance in rats fed a high-fat diet. Am J Physiol Endocrinol Metab (2009) 296(5):E1003–12. 10.1152/ajpendo.90377.2008 19116375 PMC2681305

[60] Baker RG Hayden MS Ghosh S NF-κB, inflammation, and metabolic disease. Cell Metab (2011) 13(1):11–22. 10.1016/j.cmet.2010.12.008 21195345 PMC3040418

[61] Purkayastha S Zhang G Cai D Uncoupling the mechanisms of obesity and hypertension by targeting hypothalamic IKK-[beta] and NF-[kappa] B. Nat Med (2011) 17(7):883–7. 10.1038/nm.2372 21642978 PMC3134198

[62] Clarke JR e Silva NML Figueiredo CP Frozza RL Ledo JH Beckman D Alzheimer-associated Aβ oligomers impact the central nervous system to induce peripheral metabolic deregulation. EMBO Mol Med (2015) 7(2):190–210. 10.15252/emmm.201404183 25617315 PMC4328648

[63] Qin LM Bouchard R Pugazhenthi S. Regulation of cyclic AMP response element-binding protein during neuroglial interactions. J Neurochem (2016) 136(5):918–30. 10.1111/jnc.13497 26677139

[64] Wang R Chen S Liu Y Diao S Xue Y You X All-trans-retinoic acid reduces BACE1 expression under inflammatory conditions via modulation of nuclear factor κB (NFκB) signaling. J Biol Chem (2015) 290(37):22532–42. 10.1074/jbc.M115.662908 26240147 PMC4566228

[65] Troutwine BR Ghezzi A Pietrzykowski AZ Atkinson NS. Alcohol resistance in *Drosophila* is modulated by the Toll innate immune pathway. Genes Brain Behav (2016) 15(4):382–94. 10.1111/gbb.12288 26916032 PMC4991213

[66] Zou J Crews F. CREB and NF-kappaB transcription factors regulate sensitivity to excitotoxic and oxidative stress induced neuronal cell death. Cell Mol Neurobiol (2006) 26:385–405. 10.1007/s10571-006-9045-9 16633891 PMC11520752

[67] Zou J Crews F Induction of innate immune gene expression cascades in brain slice cultures by ethanol: key role of NF-κB and proinflammatory cytokines. Alcohol Clin Exp Res (2010) 34(5):777–89. 10.1111/j.1530-0277.2010.01150.x 20201932

[68] Shah SA Yoon GH Kim MO Protection of the developing brain with anthocyanins against ethanol-induced oxidative stress and neurodegeneration. Mol Neurobiol (2015) 51(3):1278–91. 10.1007/s12035-014-8805-7 24997566

[69] Mander BA Marks SM Vogel JW Rao V Lu B Saletin JM β-amyloid disrupts human NREM slow waves and related hippocampus-dependent memory consolidation. Nat Neurosci (2015) 18(7):1051–7. 10.1038/nn.4035 26030850 PMC4482795

[70] Narasimamurthy R Hatori M Nayak SK Liu F Panda S Verma IM Circadian clock protein cryptochrome regulates the expression of proinflammatory cytokines. Proc Natl Acad Sci U S A (2012) 109(31):12662–7. 10.1073/pnas.1209965109 22778400 PMC3411996

[71] Williams JA Sathyanarayanan S Hendricks JC Sehgal A Interaction between sleep and the immune response in *Drosophila*: a role for the NF-κB Relish. Sleep (2007) 30(4):389–400. 10.1093/sleep/30.4.389 17520783 PMC2884379

[72] Israel LP Benharoch D Gopas J Goldbart AD. A pro-inflammatory role for nuclear factor kappa B in childhood obstructive sleep apnea syndrome. Sleep (2013) 36(12):1947. 10.5665/sleep.3236 24293770 PMC3825445

[73] Angelo MF Aguirre A Reyes RXA Villarreal A Lukin J Melendez M The proinflammatory RAGE/NF-κB pathway is involved in neuronal damage and reactive gliosis in a model of sleep apnea by intermittent hypoxia. PLoS One (2014) 9(9):e107901. 10.1371/journal.pone.0107901 25265561 PMC4180086

[74] Black DS O’Reilly GA Olmstead R Breen EC Irwin MR. Mindfulness meditation and improvement in sleep quality and daytime impairment among older adults with sleep disturbances: a randomized clinical trial. JAMA Intern Med (2015) 175(4):494–501. 10.1001/jamainternmed.2014.8081 25686304 PMC4407465

[75] Van Den Heuvel C Thornton E Vink R. Traumatic brain injury and Alzheimer’s disease: a review. Prog Brain Res (2007) 161:303–16. 10.1016/S0079-6123(06)61021-2 17618986

[76] Jayakumar AR Tong XY Ruiz-Cordero R Bregy A Bethea JR Bramlett HM Activation of NF-κB mediates astrocyte swelling and brain edema in traumatic brain injury. J Neurotrauma (2014) 31(14):1249–57. 10.1089/neu.2013.3169 24471369 PMC4108982

[77] Katzenberger RJ Chtarbanova S Rimkus SA Fischer JA Kaur G Seppala JM Death following traumatic brain injury in *Drosophila* is associated with intestinal barrier dysfunction. Elife (2015) 4:e04790 10.7554/eLife.04790 PMC4377547 25742603

[78] Barekat A Gonzalez A Mauntz RE Kotzebue RW Molina B El-Mecharrafie N Using *Drosophila* as an integrated model to study mild repetitive traumatic brain injury. Sci Rep (2016) 6:25252 10.1038/srep25252 27143646 PMC4855207

[79] Nonaka M Chen XH Pierce JE Leoni MJ Mcintosh TK Wolf JA Prolonged activation of NF-κB following traumatic brain injury in rats. J Neurotrauma (1999) 16(11):1023–34. 10.1089/neu.1999.16.1023 10595819

[80] Lipponen A Paananen J Puhakka N Pitkänen A Analysis of post-traumatic brain injury gene expression signature reveals tubulins, Nfe2l2, Nfkb, Cd44, and S100a4 as treatment targets. Sci Rep (2016) 17(6):31570 10.1038/srep31570 PMC4987651 27530814

[81] Gupta RK Prasad S. Age-dependent alterations in the interactions of NF-κB and N-myc with GLT-1/EAAT2 promoter in the pericontusional cortex of mice subjected to traumatic brain injury. Mol Neurobiol (2016) 53(5):3377–88. 10.1007/s12035-015-9287-y 26081154

[82] Minegishi Y Haramizu S Misawa K Shimotoyodome A Hase T Murase T. Deletion of nuclear factor-κB p50 upregulates fatty acid utilization and contributes to an anti-obesity and high-endurance phenotype in mice. Am J Physiol Endocrinol Metab (2015) 309(6):E523–33. 10.1152/ajpendo.00071.2015 26173458

[83] Lovatel GA Elsner VR Bertoldi K Vanzella C Moysés Fdos S Vizuete A Treadmill exercise induces age-related changes in aversive memory, neuroinflammatory and epigenetic processes in the rat hippocampus. Neurobiol Learn Mem (2013) 101:94–102. 10.1016/j.nlm.2013.01.007 23357282

[84] Botezelli JD Coope A Ghezzi AC Cambri LT Moura LP Scariot PP Strength training prevents hyperinsulinemia, insulin resistance, and inflammation independent of weight loss in fructose-fed animals. Sci Rep (2016) 6:31106. 10.1038/srep31106 27487746 PMC4973231

[85] Ropelle ER Flores MB Cintra DE Rocha GZ Pauli JR Morari J IL-6 and IL-10 anti-inflammatory activity links exercise to hypothalamic insulin and leptin sensitivity through IKKβ and ER stress inhibition. PLoS Biol (2010) 8(8):e1000465 10.1371/journal.pbio.1000465 20808781 PMC2927536

[86] Hackler L Jr Ózsvári B Gyuris M Sipos P Fábián G Molnár E The curcumin analog C-150, influencing NF-κB, UPR and Akt/Notch pathways has potent anticancer activity in vitro and in vivo. PLoS One (2016) 11(3):e0149832. 10.1371/journal.pone.0149832 26943907 PMC4778904

[87] Hallahan B Ryan T Hibbeln JR Murray IT Glynn S Ramsden CE Efficacy of omega-3 highly unsaturated fatty acids in the treatment of depression. Br J Psychiatry (2016) 209(3):192–201. 10.1192/bjp.bp.114.160242 27103682 PMC9406129

[88] St-Laurent-Thibault C Arseneault M Longpre F Ramassamy C. Tyrosol and hydroxytyrosol two main components of olive oil, protect N2a cells against amyloid-β-induced toxicity. involvement of the NF-κB signaling. Curr Alzheimer Res (2011) 8(5):543–51. 10.2174/156720511796391845 21605049

[89] Godbout JP Berg BM Krzyszton C Johnson RW α-Tocopherol attenuates NF-κB activation and pro-inflammatory cytokine production in brain and improves recovery from lipopolysaccharide-induced sickness behavior. J Neuroimmunol (2005) 169(1):97–105. 10.1016/j.jneuroim.2005.08.003 16146653

[90] Gold PW Licinio J Pavlatou MG Pathological parainflammation and endoplasmic reticulum stress in depression: potential translational targets through the CNS insulin, klotho and PPAR-γ systems. Mol Psychiatry (2013) 18(2):154–65. 10.1038/mp.2012.167 23183489 PMC10064987

[91] Wang J Bi W Cheng A Freire D Vempati P Zhao W Targeting multiple pathogenic mechanisms with polyphenols for the treatment of Alzheimer’s disease-experimental approach and therapeutic implications. Front Aging Neurosci (2014) 14(6):42 10.3389/fnagi.2014.00042 PMC3954102 24672477

[92] Capiralla H Vingtdeux V Zhao H Sankowski R Al-Abed Y Davies P Resveratrol mitigates lipopolysaccharide- and Aβ-mediated microglial inflammation by inhibiting the TLR4/NF-κB/STAT signaling cascade. J Neurochem (2012) 120(3):461–72. 10.1111/j.1471-4159.2011.07594.x 22118570 PMC3253186

[93] Wentzell J Kretzschmar D. Alzheimer’s disease and tauopathy studies in flies and worms. Neurobiol Dis (2010) 40(1):21–8. 10.1016/j.nbd.2010.03.007 20302939 PMC2894260

[94] Valerio A Boroni F Benarese M Sarnico I Ghisi V Bresciani LG NF-κB pathway: a target for preventing β-amyloid (Aβ)-induced neuronal damage and Aβ42 production. Eur J Neurosci (2006) 23(7):1711–20. 10.1111/j.1460-9568.2006.04722.x 16623827

[95] Sung S Yang H Uryu K Lee EB Zhao L Shineman D Modulation of nuclear factor-κB activity by indomethacin influences Aβ levels but not Aβ precursor protein metabolism in a model of Alzheimer’s disease. Am J Pathol (2004) 165(6):2197–206. 10.1016/S0002-9440(10)63269-5 15579461 PMC1618710

[96] Medeiros R Kitazawa M Passos GF Baglietto-Vargas D Cheng D Cribbs DH Aspirin-triggered lipoxin A 4 stimulates alternative activation of microglia and reduces Alzheimer disease-like pathology in mice. Am J Pathol (2013) 182(5):1780–9. 10.1152/ajpendo.00071.2015 23506847 PMC3644736

